# Comparative Evaluation of Microbiota Engraftment Following Fecal Microbiota Transfer in Mice Models: Age, Kinetic and Microbial Status Matter

**DOI:** 10.3389/fmicb.2018.03289

**Published:** 2019-01-14

**Authors:** Tiphaine Le Roy, Jean Debédat, Florian Marquet, Carla Da-Cunha, Farid Ichou, Michèle Guerre-Millo, Nathalie Kapel, Judith Aron-Wisnewsky, Karine Clément

**Affiliations:** ^1^NutriOmics Team, INSERM, ICAN, Sorbonne Université, Paris, France; ^2^ICANalytics Facility Core, Institut de Cardiométabolisme et Nutrition (ICAN), Paris, France; ^3^Department of Functional Coprology, Pitié-Salpêtrière Hospital, Assistance Publique-Hôpitaux de Paris, Paris, France; ^4^Department of Nutrition, CRNH Ile de France, Pitié-Salpêtrière Hospital, Assistance Publique Hôpitaux de Paris, Paris, France

**Keywords:** microbiota, antibiotics, germ-free animals, polyethylene glycol, SPF, FMT fecal microbiota transplant, weaning (severage), adult

## Abstract

The intestinal microbiota and its functions are intricately interwoven with host physiology. Colonizing rodents with donor microbiota provides insights into host-microbiota interactions characterization and the understanding of disease physiopathology. However, a better assessment of inoculation methods and recipient mouse models is needed. Here, we compare the engraftment at short and long term of genetically obese mice microbiota in germ-free (GF) mice and juvenile and adult specific pathogen free (SPF) mice. We also tested the effects of initial microbiota depletion before microbiota transfer. In the present work, donor microbiota engraftment was better in juvenile SPF mice than in adult SPF mice. In juvenile mice, initial microbiota depletion using laxatives or antibiotics improved donor microbiota engraftment 9 weeks but not 3 weeks after microbiota transfer. Microbiota-depleted juvenile mice performed better than GF mice 3 weeks after the microbiota transfer. However, 9 weeks after transfer, colonized GF mice microbiota had the lowest Unifrac distance to the donor microbiota. Colonized GF mice were also characterized by a chronic alteration in intestinal absorptive function. With these collective results, we show that the use of juvenile mice subjected to initial microbiota depletion constitutes a valid alternative to GF mice in microbiota transfer studies.

## Introduction

The microorganisms that inhabit the gastrointestinal tract of mammals, termed the gut microbiota, are critical in host physiology regulation by influencing metabolism (Bäckhed et al., 2004), immunity/inflammation (Cebra, 1999), aging (Smith et al., 2017), and behavior (Leclercq et al., 2017). Whereas observational studies provide initial evidence of an association between gut microbiota composition and host pathophysiology, moving from association to causation often requires microbiota transplantation. As such, transferring host phenotypes through intestinal microbiota transplantation is indeed critical to demonstrate the causative impact of microbiota on the phenotype of interest, and this strategy is commonly used in the study of different diseases (Le Roy et al., 2013; Chassaing et al., 2015; Dey et al., 2015; Nagao-Kitamoto et al., 2016).

Despite the numerous studies employing microbiota transplantation in animal models, the methods are highly debated and there is no scientific consensus on the best methodology. Gut microbiota transfer can be achieved by co-housing donor and recipient mice or through gavaging recipient mice with donor feces or digestive content. Co-housing is an efficient microbiota transfer strategy to prove the contributing role of gut microbiota in several physiological and pathological processes (Elinav et al., 2011; Singh et al., 2018). However, apart from transferring the microbiota of a transgenic mouse to a wild mouse fed a similar diet, this strategy lacks broad applicability. For example, co-housing cannot be performed for microbiota transfer from human to mouse (Llopis et al., 2016) or when donor and recipient mice are fed different diets (Anhê et al., 2018). When performing microbiota transfer by force-feeding recipient mice with the microbiota of the donor, differences first lie in the preparation and the potential conservation of the inoculum. Donor inoculum can be transferred to recipient animal models immediately after collection (Le Roy et al., 2013), frozen without prior preparation (Tremaroli et al., 2015), or frozen with a cryoprotectant (Llopis et al., 2016). Secondly, the recipient rodent model varies substantially across the literature. Many studies use germ-free (GF) rodents for microbiota transfer (Ziȩtak et al., 2016) while others use conventionally raised animals with or without microbiota depletion before the transfer (Gregory et al., 2015; Ferrere et al., 2017). Lastly, the frequency and the length of inoculum administration range from a single administration to twice a week for several weeks (Hintze et al., 2014; Ferrere et al., 2017; Wrzosek et al., 2018). These experimental strategies have more or less been documented regarding the efficacy of microbiota transfer, which can be defined as (i) establishment of a high proportion of inoculum’s bacterial taxa in recipients, (ii) relative abundances of bacterial taxa in the recipients as similar as possible to that of the inoculum and (iii) the removal of a high proportion of endogenous bacterial taxa in case of non-GF recipients.

Germ-free rodents are the most frequently used recipient model as their intestines, which are devoid of endogenous microorganisms, theoretically constitute a “blank slate” due to the absence of ecological niche competition. However, the phenotype characterization of GF and former GF mice counterbalance this assumption. The absence of microbiota during gestation and weaning alters physiology as evidenced by GF rodents exhibiting immune system deficits, metabolic alterations with reduced nutrient absorption, and different social behavior (Desbonnet et al., 2013; Smith et al., 2017). Importantly, these alterations are not entirely reversed by the conventionalizing these animals through the colonization of gut microbiota as conventionalized GF mice remain susceptible to some diseases such as asthma and intestinal inflammation. For example, colonization with a complex microbiota in post-weaning (3–4 week old) GF mice fails to decrease the 1000- to 10,000-fold increase in plasma IgE levels (Cahenzli et al., 2013) and a 3- to 5-fold increase in invariant natural killer T cells in the lung and the colon lamina propria of these animals (Olszak et al., 2012). Conversely, conventionalized mice have a lower number of helper T cells in the intestine lamina propria compared to specific pathogen free (SPF) or conventionally raised animals (Chung et al., 2012). Finally, conventionalization results in a selective enrichment of Bacteroidetes at the expense of Firmicutes (Turnbaugh et al., 2008; Wos-Oxley et al., 2012). Yet, the detailed dynamics of microbial taxa engraftment has not been well explored.

To overcome the above cited limits of GF mice models, gut microbiota engraftment in conventional or SPF mice has also been evaluated several times over the past few years (Manichanh et al., 2010; Wos-Oxley et al., 2012; Hintze et al., 2014; Ericsson et al., 2017; Ji et al., 2017; Staley et al., 2017; Wrzosek et al., 2018). In an effort to reproduce the absence of ecological niche competition similar as found in the GF model, most studies have depleted the recipient initial intestinal microbiota with broad-spectrum antibiotics. Initial studies demonstrated vancomycin or ciprofloxacin administered before microbiota transfer does not increase the number of successfully implanted bacterial taxa in comparison with non-treated animals (Manichanh et al., 2010; Wos-Oxley et al., 2012). However, broad-spectrum antibiotic combinations such as ampicillin, vancomycin, neomycin and metronidazole or sequential administration of ampicillin + cefoperazone + clindamycin and ertapenem + neomycin + vancomycin are more efficient than a single antibiotic to improve microbiota engraftment (Hintze et al., 2014; Staley et al., 2017). Despite efficient initial microbiota depletion using a mixture of four antibiotics (ampicillin, vancomycin, neomycin, and metronidazole) in drinking water, inoculum engraftment is better when the richness of the donor microbiota is higher than the richness of the recipient’s microbiota before antibiotic treatment (Ericsson et al., 2017). Moreover, conventional mice whose microbiota has been depleted using antibiotics has limitations that include antibiotic-induced alterations in mitochondrial function, bacterial translocation, and mild inflammation (Grasa et al., 2015; Morgun et al., 2015; Knoop et al., 2016). Furthermore, questions remain about the impact of residual antibiotics on the engraftment of inoculated bacteria. Bowel cleansing with laxative-based approaches, such as polyethylene glycol (PEG) also give discordant results, likely due to differences in the concentration and volume administered (Ji et al., 2017; Wrzosek et al., 2018). Although promising, this strategy has not yet been properly compared to GF and antibiotic-treated conventional recipients.

While the microbiota shapes host immunity, the immune system shapes the gut microbiota as well through the secretion of IgA and antimicrobial peptides (Kubinak et al., 2015). The secretion of IgA and antimicrobial peptides in the lumen is minimal at birth but is augmented within days after weaning (van der Heijden et al., 1989; Cash et al., 2006; Burger-van Paassen et al., 2012; Lindner et al., 2015). Provided this finding, the weaning period might be an optimal window to engraft microbiota of interest in conventionally raised mice, as the immune system underwent a normal development but does not yet exert significant pressure on the microbiota. We herein aimed to decipher how recipient microbial status, age, and microbiota depletion with different pre-treatments determine the engraftment of a transplanted gut microbiota. We evaluated the engraftment of an exogenous microbiota, the removal of the endogenous microbiota and the resulting intestinal ecosystem structure, and dynamics in different recipient models. First, microbiota engraftment was evaluated by the overall similarity between the inoculum and the recipients’ microbiota. Then, we compared the number the colonizing bacterial taxa across recipient mouse models as well as taxa not originating from the inoculum and but detected in recipient mice. Finally, we compared the relative abundance of bacterial taxa that were common to the inoculum and the recipients’ microbiota.

## Materials and Methods

### Mice

Three-week old and 8-week old specific-pathogen-free (SPF) male C57BL/6J mice were obtained from Charles River Laboratories (France). Eight-week old GF male C57BL/6J mice were obtained from the CDTA (Département de Cryopréservation, Distribution, Typage et Archivage animal, Centre National de la Recherche Scientifique, France). Twelve-week old male B6.V-Lep^ob^/JRj (*Lep^ob^*) were reared from heterozygous breeding pairs by our local mouse phenotyping core facility (Preclin-ICAN, Institute of Cardiometabolism and Nutrition, Paris). Mice were housed in groups and maintained on a 12-hour light-dark cycle with *ad libitum* access to water and standard chow diet (reference A04-10, Safe-Diets). Mice were housed in Individually Ventilated Cages (IVC). Food and bedding were irradiated at 10 kGy. Drinking water was filtered and treated with 2 ppm chlorine. Body weight and food intake were monitored once or twice a week. Mice were systematically handled under a laminar flow cabinet, gloves were changed between the handling of each group, and all instruments and working area were disinfected with hydrogen peroxyde 3% vol/vol.

### Inoculum Preparation for Fecal Microbiota Transplantation

Three twelve-week old male *Lep^ob^* were used as fecal microbiota donors. These mice were acclimated in the facility during 1 week before the beginning of the experiment. In the morning, feces from the donor mice were collected in sterile containers and immediately diluted (1:20 w/vol) in sterile, anoxic Ringer buffer containing 1 g/L L-cysteine as a reducing agent. An aliquot of inoculum was snap frozen in liquid nitrogen and stored at -80°C for subsequent bacterial composition analysis.

### Treatments and Fecal Microbiota Transplantation

**FIGURE 1:**
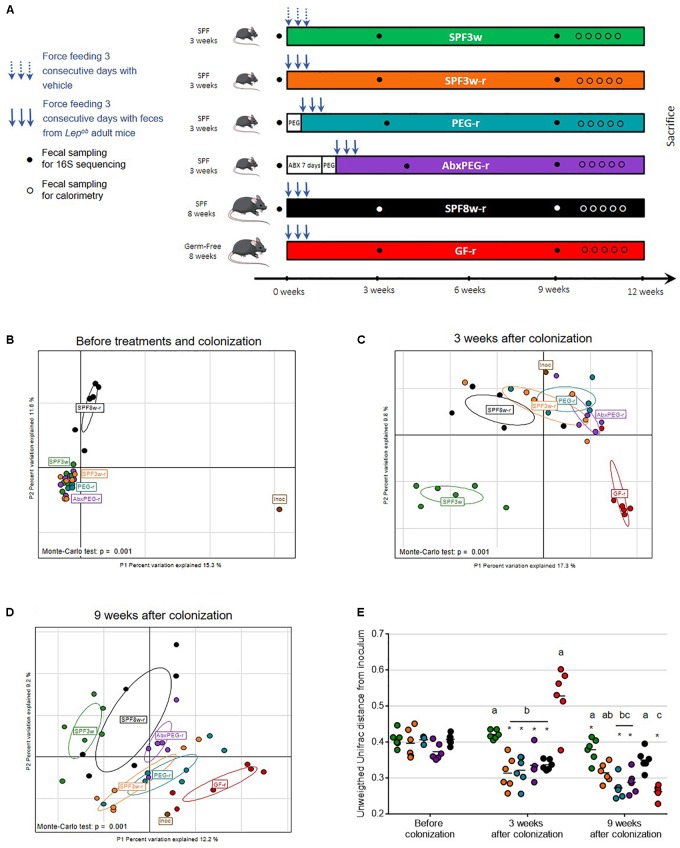
Impact of recipient age, microbiological status, and pre-treatment on fecal microbiota transfer. **(A)** Experimental design. **(B–D)** Interclass principal component analysis performed based on OTUs (operational taxonomic units) abundance before **(B)**, three weeks **(C)**, and 9 weeks after fecal microbiota transfer **(D).** Mice microbiota compositions were clustered and the center of gravity computed for each group. The *p*-value of the link between recipients’ groups and OTUs abundance was calculated using a Monte Carlo test (999 replicates). **(E)** Unweighted Unifrac distance between the inoculum and recipients bacterial communities. For all figures: Inoc, inoculum; SPF, Specific pathogen free; GF, Germ-free; PEG, polyethylene glycol; Abx, antibiotics; 3w, 3-week old; 8w, 8-week old; r, recipient. Groups with differing letters are significantly different (adjusted *p* < 0.05). ^∗^At a time-point indicates a significant change since the previous time-point.

Mice were divided in six groups of six mice. Colonization was achieved by intragastric gavage with 200 μl of inoculum once per day for three consecutive days. The experimental design and the different groups are summarized in Figure 1A. The control group was comprised of 3-week old SPF mice (SPF3w) gavaged with Ringer’s solution containing L-cysteine. A group of 3-week old and a group of 8-week old SPF mice were inoculated without prior treatment and were respectively designated as SPF 3-week recipients (SPF3w-r) and SPF 8-week recipients (SPF8w-r). A group of 3-week old SPF mice was subjected to a bowel cleansing with 1.2 ml of PEG solution before the inoculation of *Lep^ob^* fecal microbiota and is later referred as PEG-recipients (PEG-r). PEG solution contained PEG 3350 (77.5 g/L), sodium chloride (1.9 g/L), sodium sulfate (7.4 g/L), potassium chloride (0.98 g/L) and sodium bicarbonate (2.2 g/L) diluted in sterile tap water and was divided in five equal doses that were administered by oral gavage at 30 min intervals after a 2 h fast. *Lep^ob^* fecal microbiota transfer was performed 6 h after the last PEG administration. The endogenous intestinal microbiota of a last group of 3-week old SPF mice was depleted by gavage with broad spectrum antibiotics over 7 days (Reikvam et al., 2011). The antibiotics solution consisted of ampicillin, neomycin, metronidazole and vancomycin diluted in sterile water. Mice received 200 mg/kg of ampicillin, neomycin and metronidazole and 100 mg/kg of vancomycin once a day. After 7 days, the residual luminal microbiota and the antibiotics were flushed out using 1.5 ml of PEG solution. The PEG solution and the inoculum were prepared and administered as previously described and provided 24 h after the last antibiotics gavage. This group is later referred as antibiotics and PEG-treated recipients (AbxPEG-r). Eight-week old GF mice were inoculated with *Lep^ob^* fecal microbiota immediately after the opening of their sterile shipping container. This group is later referred as Germ-Free-recipients (GF-r). Mice were transferred into clean cages several times over the course of bowel cleansing treatment and *Lep^ob^* fecal microbiota administration.

### Intestinal Energy Absorption

Nine weeks after the beginning of the experiment, mice were placed on metal grids within their cages and feces were collected every 24 h for 5 days. During the same time, the food intake was monitored. The feces were dried at 40°C for 24 h and weighed. Total energy of the diet and the feces was determined by bomb calorimetry (reference C200, IKA, United States). The net intestinal energy absorption capacity is expressed as a percentage of total energy ingested and represents the proportion of ingested energy that was not recovered in feces.

### Short Chain Fatty Acid Concentrations in Cecal Content

Sample preparation was adapted from Qiu et al. (2007) and Zheng et al. (2013). Extraction protocols were always carried out close as possible to 4°C to prevent loss of SCFA species. Approximatively 20 mg (20.2–22.8 mg) of cecal content was mixed with 500 μl of 0.005 M aqueous NaOH in a precellys system with an internal standard mix composed of acetic acid-d_3_ (300 μM), propionic-d_2_ (100 μM) and butyrate-^13^C_2_ (300 μM). Three hundred μl of homogenates were transferred to glass tubes (10 ml) and 500 μl of propanol/pyridine mix (3:2, v/v) was added. Two successive volumes of 50 μl of PCF were added after vortex and sonication steps during 1 min to ensure the total derivation of SCFA species. Derivate extraction procedure was performed by adding 500 μl of hexane to the sample mixture. The reactive mixture were briefly vortexed and sonicated and centrifuged for 5 min at 2000 *g* and 4°C. Two hundred μl of organic layer were transferred to glass vials and analyzed by GC-MS.

One μl of derivatized samples were analyzed using a Trace 1310 gas chromatograph coupled to an ISQ LT mass spectrometer (Thermo Scientific, Waltham, MA, United States). Derivatized samples was injected into a DB-5MS capillary column coated with 5% Phenyl-Arylene/95% Dimethylpolysiloxane (50 m × 250 mm i.d., 0.25 μm film thickness; Agilent technologies, J&W GC columns, Les Ulis, France) in 20:1 split mode. A constant flowrate of 1 ml.min-1 of Helium gas was used in the column. Inlet temperatures, ion source and MS transfer line temperatures were set to 260, 230, and 290°C, respectively. Electron impact energy (70 eV) and selected ion monitoring (SIM) mode were used to measure SCFA derivates with a detection delay time set to 4.5 min. Briefly, for the oven settings, the oven temperature was initially held at 50°C for 1.5 min and then, increased to 70°C with 8°C/min rates. A ramp of 6°C/min until 85°C was applied. Temperature was raised firstly to 110°C with a 22°C/min rate and then to 120°C with 12°C/min rate. Finally, 300°C was reached with a 125°C/min rate and held for 3 min.

### Microbiota Composition Analysis

Feces were collected when mice arrived in the animal facility, three weeks after the colonization with *Lep^ob^* fecal microbiota, and nine weeks after the beginning of the experiment. For this purpose, mice were placed in sterile containers and allowed to defecate. Pellets were immediately collected and snap frozen in liquid nitrogen. Fecal DNA was extracted as previously described (Godon et al., 1997).

Amplification and sequencing were performed at Diversigen, Inc., (Houston, TX, United States). The raw data of libraries generated during this study is publicly available at the Sequence Read Archive (SRA) portal of NCBI under accession number PRJNA508481. Briefly, 16S rRNA gene V4 region was amplified using 517F (5′-GCCAGCAGCCGCGCTAA-3′) and 798R (5′-184 AGGGTATCTAATCCT-3′) primers. Amplicons were sequenced using Illumina MiSeq sequencing (2 × 250 bp). Sequences were merged using an overlap of at least 50 bps, then merged sequences were stripped from primer sequences and oriented identically. Sequences were then clustered into Operational Taxonomic Units (OTUs) at the 97% identity level and taxonomies were determined using USEARCH (Edgar, 2010) and SILVA(v4) database (Quast et al., 2013). Data were normalized using a rarefaction cutoff of 5,959 reads. Taxonomic binning and Unifrac distances calculations were performed using Rhea scripts (Lozupone and Knight, 2005; Lagkouvardos et al., 2017) and R 3.3.2 program.

### Statistical Analyses

Results are represented as mean ± SEM. Comparisons between groups were performed by Kruskal–Wallis test followed by pairwise multiple comparisons using Dunn procedure. Adjusted *p*-values were obtained using Benjamini–Hochberg correction. Comparisons of different timepoints were performed by repeated measures ANOVA followed by Benjamini–Hochberg correction. Adjusted *p*-values < 0.05 being considered statistically significant. All statistical tests were performed using R 3.3.2 program. Principal component analyses (PCA) were performed using R program and ade4 package. Interclass PCA were computed and statistically assessed by a Monte Carlo rank test to observe their net effect on the scattering of the microbiota of different mice. Venn diagrams and Heatmaps were generated using R program and VennDiagram and Superheat packages, respectively. Bar plots, scatter dot plots, box and whiskers plots were generated using GraphPad Prism 7.0.

## Results

### Experimental Strategy

*C57Bl6-Lep^ob^* mice were chosen as intestinal microbiota donor since it has been well described that their intestinal microbiota composition substantially differs from those of wild–type mice on the same genetic background (Ley et al., 2005), thus facilitating the examination of microbiota composition changes following colonization in various settings.

As shown in Figure 1A, six groups of mice were used, of which five were inoculated with *Lep^ob^* intestinal microbiota. To test the hypothesis that younger age favors exogenous microbiota engrafment, 3-week old and 8-week old SPF mice were colonized and referred as SPF3w-recipients (SPF3w-r) and SPF8w-recipients (SPF8w-r). Assuming that using mice during the weaning period (SPF3w-r) would be a favorable window for engraftment, we tested whether additional methods of bowel cleansing would add further in engraftment efficiency. We depleted the intestinal microbiota of 3-week old SPF mice with either efficient bowel cleansing with PEG or 7 days antibiotics treatment (ampicillin, vancomycin, neomycin, and metronidazole) followed by bowel cleansing with PEG. These groups are later referred as PEG-recipients (PEG-r) and antibiotics-PEG-recipients (AbxPEG-r). To compare these recipient models to the commonly published reference model for microbiota transfer, we added a group of 8-week old germ-free recipients (GF-r). Except for control SPF3w mice, all groups were inoculated three consecutive days with a suspension of fresh *Lep^ob^* feces. Controls were gavaged with the same volume of the vehicle solution. To assess similarities and dissimilarities between fecal communities, we used 16S rRNA gene sequencing of the V3-V4 region for the inoculum, the SPF mice at their arrival, and all groups 3 weeks post-inoculation and 9 weeks after the experiment start.

### Age and to a Lesser Extent Pretreatments Determine Gut Microbial Community Reshaping

Sequences of the 16S rRNA gene were clustered into Operational Taxonomic Units (OTUs), or phylotypes, using a 97% identity level. Interclass PCA based on the OTUs abundance showed that the microbial composition of the inoculum greatly differed from all SPF mice before colonization (Figure 1B). Before microbiota transfer, 3-week-old and 8-week-old mice’s microbiota clustered separately and 8-week old mice’s microbiota showed greater dispersion. Three weeks after the oral administration of the *Lep^ob^* fecal slurry, all SPF recipients clustered closely with the inoculum regardless of age or method of microbiota depletion (Figure 1C), supported by significantly lower unweighted UniFrac distances from the inoculum (Figure 1E). Microbiological status (GF vs. SPF) had an effect on microbiota composition at 3 weeks post-innoculation (Figure 1C). However, 6 weeks later, the microbiota of 3-week old SPF and GF recipients clustered more closely with the inoculum than 8-week old recipients (Figure 1D). Throughout the study, non-inoculated mice’s microbiota remained distant from the inoculum microbiota communities as expected. These collective results were supported by significantly lower unweighted UniFrac distances from the inoculum (Figure 1E). In addition, SPF3w-r and SPF8w-r bacterial communities remained at the same unweighted UniFrac distance while pretreated counterparts further converged toward the inoculum. Comparison of PEG-r and AbxPEG-r groups showed that microbiota depletion with broad-spectrum antibiotics did not improve the resemblance with the inoculum as compared to a simple bowel cleansing with laxatives. SPF3w microbiota was slightly closer to the inoculum 9 weeks after the beginning of the experiment than at 3 weeks, supported by a decrease of mean unweighted UniFrac distance from 0.423 to 0.379 (*p* = 0.001).

### Germ-Free Status Delays Transferred Microbiota Establishment

Three weeks after the microbiota transfer, the established fecal bacterial community in GF recipients was the most distant from the inoculum in respect to clustering based on OTUs abundance and unweighted UniFrac distance (Figures 1C,E). Intriguingly, GF-r bacterial communities were even further from inoculum than non-inoculated SPF3w mice, with a mean UniFrac distance of 0.528 vs. 0.423 (Figures 1C,E). This observation was supported by a clearly altered microbiota composition of the GF-r three weeks after colonization, which was characterized by low bacterial richness (76.8 vs. 180 for the inoculum) and a significant switch of the relative abundance of dominant bacterial phyla. Specifically, Firmicutes were underrepresented with a relative abundance of 25.3% (vs. 57.3% in the inoculum) while other phyla bloomed: Bacteroidetes reached 55% (vs. 39.9% in the inoculum), Actinobacteria 7.96% (vs. 0.59% in the inoculum) and Verrucomicrobia 6.66% (vs. 0.32 % in the inoculum) (Supplementary Figures 1A–E). These differences disappeared six weeks later as GF-r microbiota clustered with the inoculum and had the lowest unweighted UniFrac distance from the inoculum, although there was no statistical difference with the two pre-treated SPF3w recipients groups: PEG-r and AbxPEG-r (Figures 1D,E).

### Age Does Not Influence the Engraftment of Exogenous Phylotypes in SPF Mice

**FIGURE 2:**
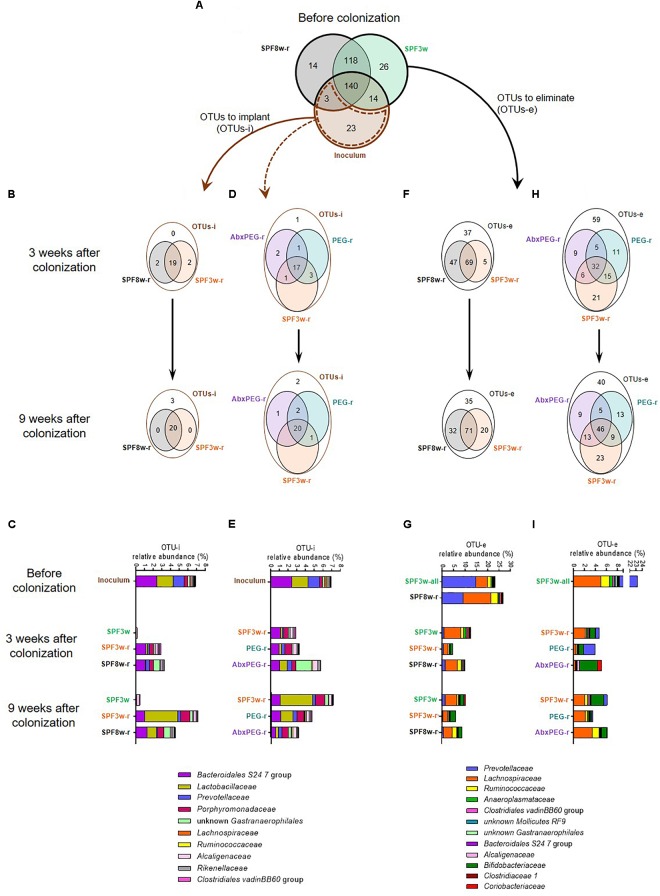
Age and pre-treatment influence the number and relative abundance of OTUs that do or do not successfully colonize recipient mice and OTUs that are or are not successfully eliminated. **(A)** Venn diagram representing the overlap of OTUs in the inoculum and in 3 and 8 weeks old SPF mice before the fecal microbiota transfer. **(B)** Effect of age on the implantation of exogenous bacteria in recipient mice. Venn diagrams representing the OTUs to implant (OTUs-i, i.e., OTUs that were present in the inoculum but not present either in 3-week or 8-week old SPF mice before colonization) in SPF3w, SPF3w-r and SPF8w-r groups 3 and 9 weeks after colonization. **(C)** Relative abundance of the bacterial families corresponding to the OTUs-i in the inoculum and in SPF3w, SPF3w-r and SPF8w-r groups 3 and 9 weeks after colonization. **(D)** Effect of pre-treatment on the implantation of exogenous OTUs in 3-week old SPF mice. Venn diagrams representing the overlap of the OTUs-i in SPF3w-r, PEG-r and AbxPEG-r groups 3 and 9 weeks after colonization. **(E)** Relative abundance of the bacterial families corresponding to the OTUs-i in the inoculum and in SPF3w-r, PEG-r and AbxPEG-r groups 3 and 9 weeks after colonization. **(F)** Effect of age on the removal of endogenous bacteria in recipient mice. Venn diagram representing the OTUs to eliminate (OTUs-e; i.e., OTUs that were present in the fecal microbiota of 3 and 8-week old SPF mice before colonization but not in the inoculum) in SPF3w, SPF3w-r and SPF8w-r groups 3, and 9 weeks after colonization. **(G)** Relative abundance of the bacterial families corresponding to the OTUs-e in SPF3w, SPF3w-r and SPF8w-r groups 3, and 9 weeks after colonization. **(H)** Effect of laxative and antibiotic pre-treatments on the removal of endogenous bacteria in 3-week old SPF mice. Venn diagram representing the overlap of the OTUs-e in SPF3w-r, PEG-r and AbxPEG-r groups 3 and 9 weeks after colonization. **(I)** Relative abundance of the bacterial families corresponding to the OTUs-e in the inoculum and in SPF3w-r, PEG-r and AbxPEG-r groups 3 and 9 weeks after colonization.

To further determine which *Lep^ob^* phylotypes were established in the different recipient models, we counted the OTUs that were present in the inoculum but not in SPF mice before inoculation, (OTUs to implant; OTUs-i). The dynamics of engraftment were also examined. Twenty-three OTUs-i were listed and represented 6.96% of the inoculum. After taxonomic assignation of these OTUs at the family level, we found that they belonged to diverse bacterial families but that the most abundant in the inoculum belonged to *Bacteroidales* S24 7 group, *Lactobacillaceae* and *Prevotellaceae* families (Figures 2A,C). The majority of these 23 OTUs-i successfully colonized in both SPF3w-r and SPF8w-r mice at 21 days (19 OTUs-i) and 9 weeks (20 OTUs-i) after inoculation (Figure 2B).

Engraftment dynamics were dependent on recipient age. While most OTUs-i were detected in SPF recipients, the relative abundance of *Lactobacillaceae* reached an abundance similar to that of the inoculum only 9 weeks after colonization and the overall abundance of OTUs-i in SPF3w-r mice resembled the inoculum more than that of SPF8w-r mice (Figure 2C and Supplementary Figure 2). OTUs-i belonging to *Ruminococcaceae* family failed to colonize SPF3w-r and SPF8w-r at the 9 week time-point.

Most of the OTUs-i were not detected in non-inoculated mice at both time-points, except three OTUs-i belonging to the *Alcaligenaceae*, *Lachnospiraceae*, and *Coriobacteriaceae* families (Figure 2C and Supplementary Figure 2). However, these OTUs-i remained at very low abundances. This indicates that individually ventilated cages are sufficient to prevent bacteria transfers between cages for at least 9 weeks provided that basic hygienic measures are applied when handling mice.

### Pre-treatments Differentially Influence Colonization by Exogenous Phylotypes in SPF Mice

Compared to SPF3w-r recipients, pre-treated groups (PEG-r and AbxPEG-r) harbored a better engraftment of *Ruminococcaceae* as 3 out of 4 instead of 1 out of 4 OTUs-i colonized PEG-r and AbxPEG-r groups (Figures 2D,E and Supplementary Figure 2). Antibiotic pre-treatment, however, prevented the implantation of *Mollicutes* RF9, delayed the implantation of *Lachnospiraceae*, and induced a transient bloom of unknown *Gastranaerophilales*.

### Young Age Favors the Removal of Endogenous Phylotypes

As the biggest challenge in the use of SPF mice for gut microbiota transfer is the presence of an endogenous microbiota that occupies the ecological niche, we listed the OTUs to eliminate (OTUs-e). This represents the OTUs that were present in the SPF mice at their arrival but absent from the inoculum. Respectively 144 and 132 OTUs-e were detected in the microbiota of 3-week-old and 8 week-old mice before the microbiota transfer (Figure 2A). We observed that 116 (80.5%) and 103 (78%) OTUs-e persisted in SPF8w-r mice 3 and 9 weeks, respectively after microbiota transfer (Figure 2F). In the SPF3w-r group, 74 (51.4%) OTUs-e persisted 3 weeks after inoculation, although it later rose to 91 (63.2%) OTUs-e 6 weeks later. However, these persistent OTUs-e represented only 4.5 and 6% of the whole microbiota in SPF3w-r mice compared to 10 and 9% in SPF8w-r recipients (Figure 2G) (*p* = 0.032 and *p* = 0.14). This indicates that endogenous microbiota is more effectively replaced by exogenous microbiota at weaning than during adulthood.

The largest proportion of the OTUs-e that were not eliminated in SPF3w and SPF8w recipients belonged to the *Lachnospiraceae* and *Bifidobacteriaceae* families (Figure 2G and Supplementary Figure 3). Most of the families corresponding to persistent OTUs-e were less abundant in SPF3w-r mice than in SPF8w-r mice, although these differences did not reach statistical significance. The total relative abundance of those OTUs-e spontaneously decreased from 23.2 to 12.4% and 9.6% in non-inoculated mice 3 and 9 weeks after the beginning of the experiment. This decrease mainly reflects a massive drop of the abundance of *Prevotellaceae*. This highlights that environmental changes resulting from the transfer from the supplier facilities to our animal facilities and not only the inoculation with *Lep^ob^* microbiota has a strong impact on microbiota composition of recipient mice.

### Pre-treatments Differentially Affect the Removal of Endogenous Phylotypes

As the replacement of the endogenous microbiota by the inoculum microbiota appeared more efficient at weaning, we sought to further improve the microbiota transfer by removing endogenous microbiota by either PEG or AbxPEG treatments. These treatments allowed the removal of 12 and 22 additional OTUs-e (43 and 37.5%) 21 days after microbiota transfer (Figure 2H). Nine weeks after the transfer, laxatives and antibiotics treatments provided similar results as in PEG-r and AbxPEG-r 18 more OTUs-e were eliminated in comparison to SPF3w-r. Remaining OTUs were binned into families in order to identify the lineages that were or were not successfully eliminated from the recipient’s microbiota.

Both PEG and AbxPEG treatments allowed the complete removal of OTUs-e belonging to the *Bacteroidales* S24-7 (Figure 2I and Supplementary Figure 4). However, only AbxPEG treatment successfully removed all unknown *Gastranaerophilales* and *Prevotellaceae*. A transient rise of *Alcaligenaceae*, *Bifidobacteriaceae* and *Coriobacteriaceae* was observed 21 days after microbiota transfer in the AbxPEG-r group, which normalized 6 weeks later. SPF3w-r and PEG-r groups were also affected by this rise of OTUs-e belonging to *Bifidobacteriaceae* family. The overall abundance of OTUs-e was similar in SPF3w-r and AbxPEG-r groups at both time points (4.6–6.1% of the microbiota) and slightly lower in PEG-r recipients (3.9 and 3.5% of the microbiota at 21 days and 9 weeks).

### GF Recipients Successfully Acquire Most of the Donor Microbial Community After 3 Weeks

**FIGURE 3:**
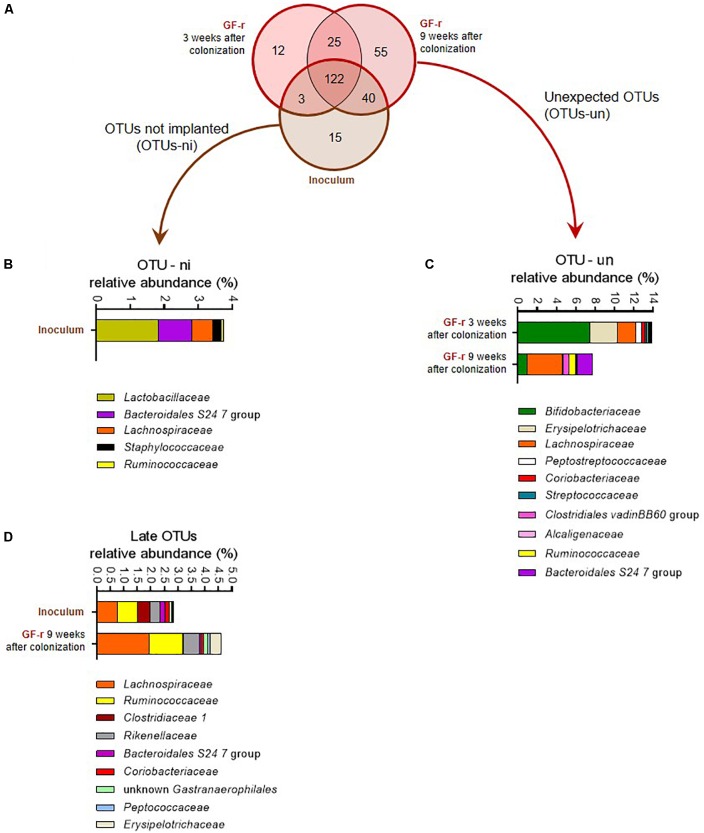
Number and relative abundance of OTUs that did or did not successfully colonize germ-free recipient mice. **(A)** Venn diagram representing the overlap of OTUs in the inoculum and in GF-r mice 3 and 9 weeks after fecal microbiota transfer**. (B)** Relative abundance of the bacterial families corresponding to the OTUs-ni (OTUs from the inoculum that were not implanted in GF-r mice) in the inoculum. **(C)** Relative abundance of the bacterial families corresponding to the OTUs that were found in GF-r mice but were not detected in the inoculum (OTUs-un). **(D)** Relative abundance of the bacterial families corresponding to the OTUs were detected in GF-r mice feces at 9 weeks but not at 3 weeks post inoculation.

Most of the OTUs present in the inoculum successfully colonized the gut of GF recipients as 125 and 162 out of the 180 OTUs-i were detected in the feces of the GF-r mice 3 and 9 weeks after the microbiota transfer (Figure 3A).

Fifteen OTUs failed to colonize the gut of GF recipients (OTUs non-implanted: OTUs-ni). Most of these OTUs-ni belonged to *Lactobacillaceae*, *Bacteroidales* S 24-7 and *Lachnospiraceae* families (Figure 3B). Yet, the summed relative abundance of OTUs-ni in the inoculum was only 3.76%, indicating that OTUs-ni represented only a minor part of the *Lep^ob^* donors’ microbiota.

Forty OTUs originating from the inoculum were detected in GF-r mice feces at 9 weeks but not at 3 weeks post inoculation. As recipients were inoculated with donors’ microbiota only three consecutive days at the beginning of the experiment, those 40 OTUs were likely present in the microbiota of GF-r mice 21 days after inoculation but below detection limits. Taxonomic binning revealed that those “late” OTUs belonged to *Lachnospiraceae*, *Ruminococcaceae* and *Rikenellaceae* families (Figure 3D), all known to be strictly anaerobic. Similar to OTUs-ni, OTUs that colonized the gut of GF mice with a delayed pattern represented only a minor proportion of the microbiota in both the inoculum and recipients (2.8 and 4.6%).

### A Significant Number of Unexpected Phylotypes Colonized GF Recipients

We detected 37 and 80 OTUs that were not detectable in the inoculum, but which were present in the microbiota of GF-r mice 3 and 9 weeks after the colonization (Figure 3A). Those unexpected OTUs reached nearly 14% of the GF-r microbiota 21 days after colonization and decreased to 7.8% 6 weeks later (Figure 3C). As in AbxPEG-r, those unexpected OTUs mainly belonged to the *Bifidobacteriaceae* family 21 days after colonization and to the *Lachnospiraceae* family 9 weeks after colonization. *Peptostreptococcaceae* and *Erysipelotrichaceae* represented a substantial part of unexpected OTUs 3 weeks after colonization but vanished at 9 weeks, while OTUs identified as *Ruminococcaceae*, *Bacteroidales* S 24-7 and *Clostridiales vadin* BB60 group appeared. Those unexpected OTUs could also be OTUs that were present in the inoculum below detection limits as well as OTUs originating from the environment that contaminated GF recipient.

### Microbiota Transfer Models Differentially Affect Gut Ecosystem Structure

**FIGURE 4:**
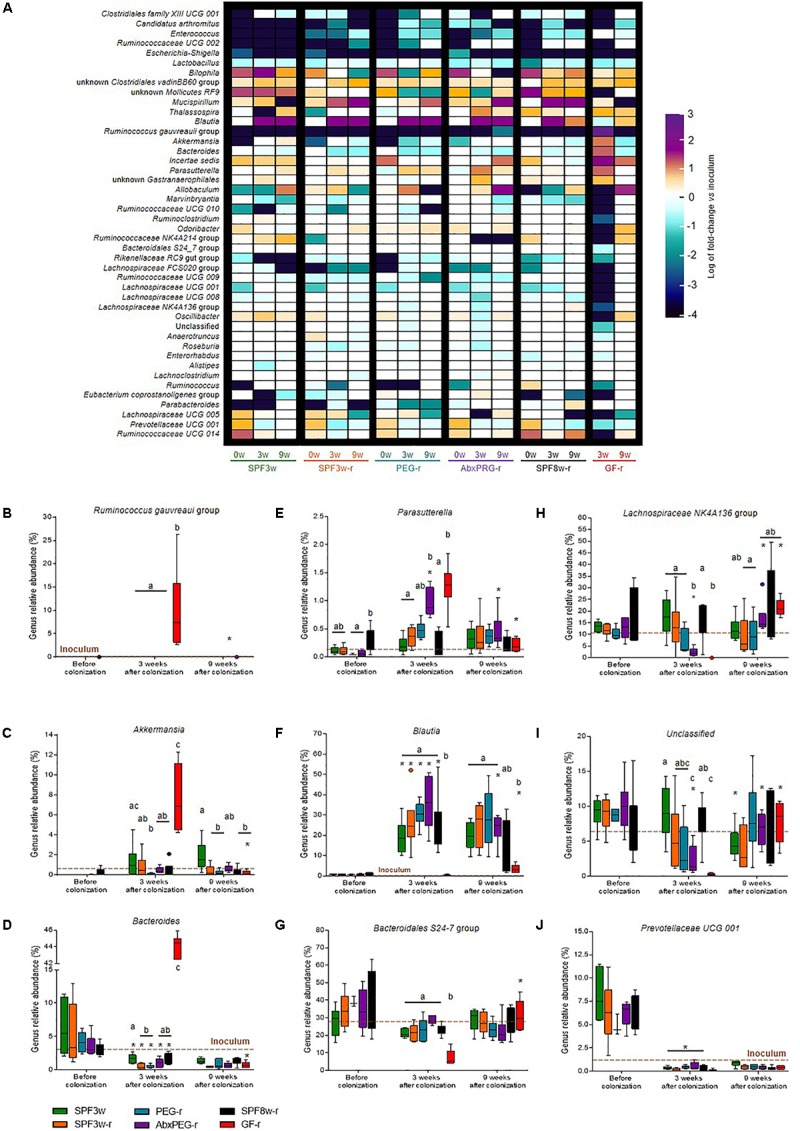
Relative abundance of bacterial genera present in the inoculum and in recipient mice. **(A)** Heatmap representing the fold change of the relative abundance of bacterial genera between the inoculum and the different mice groups at all time-points. Only the 48 most abundant genera are represented. **(B–J)** Relative abundance of *Ruminococcus gauvreaui* group **(B)**, *Akkermansia* **(C)**, *Bacteroides* **(D)**, *Parasutterella* **(E*)****, Blautia* **(F)**, *Bacteroidales* S24-7 **(G)**, *Lachnospiraceae* NK4A136 **(H)** unclassified **(I)** and *Prevotellaceae* UCG 001 **(J)** genera in all groups and time-points. A brown dotted line indicates the relative abundance in the inoculum. Groups with different letters are significantly different (adjusted *p* < 0.05). Groups with a ^∗^ at a time-point are significantly different from the previous time-point.

We further compared the proportions of shared taxa between the inoculum and recipients before microbiota transfer at all time points and between all groups. The relative abundance of the 44 most abundant shared genera was compared to the inoculum (Figure 4A). First, we observed that several genera such as *Escherichia–Shigella*, *Enterococcus*, and *Lactobacillus* genera were less abundant in all groups than in the inoculum (Supplementary Figures 5A–C). By contrast, the relative abundance of low abundance genera such as the *Clostridiales vadin* BB60 group was higher in all groups than in the inoculum. We also found that several genera had an overall similar relative abundance in the inoculum and in SPF recipients, yet were significantly higher in GF-r microbiota at the day 21 time point. For example, the group *Ruminococcus gauvreaui* reached 8.3% relative bacterial abundance in GF-r mice at 21 days while it was barely detectable in the inoculum of all other groups (Figure 4B). Likewise, the genus *Akkermansia* had a relative abundance of 6.66% in GF-r at 21 days while it comprised 0.62% of the inoculum and between 0.07 and 0.84% of the SPF recipients’ microbiota (Figure 4C). The *Bacteroides* genus was also altered in GF-r microbiota 21 days post-inoculation as it constituted 36.8% of the microbiota, and it represented only 3.9% of the inoculum and did not reached more than 2.82% of the microbiota in SPF recipients (Figure 4D). Mirroring this transient overabundance of some taxa, several other genera had a significantly lower abundance in GF-r mice 3 weeks after the colonization. Similar to the OTUs-i that successfully colonized GF recipients only at the 9 week time-point, most the under-represented genera in GF recipients 3 weeks after the microbiota transfer belonged to the *Ruminococcaceae*, *Rikenellaceae* and *Lachnospiraceae* families (Supplementary Figure 5). One of the few exceptions was the dominant group *Bacteroidales* S24-7, with a relative abundance of only 10.5% in GF recipients 21 days after colonization versus an average abundance above 20% in all other recipients groups and in the inoculum (Figure 4G). Most of these alterations did not persist at 9 weeks after the colonization.

Several genera exhibited a similar kinetic of abnormal high/low abundance at the 21 days time-point followed by a normalization at the 9 weeks time-point in SPF mice treated with antibiotics before the colonization. Unknown *Gastranaerophilales* and *Parasutterella* genera were particularly abundant in both GF-r and AbxPEG-r 21 days after colonization (Figure 4E). On the contrary, several *Ruminococcaceae* and *Lachnospiraceae* clusters as well as *Oscillibacter* genus and unclassified phylotypes were significantly lower in GF-r and AbxPEG-r groups than in other recipient groups (Figures 4H,I).

Unexpectedly, the *Blautia* genus proliferated in all SPF mice groups despite being found at very low levels both in the inoculum (0.67%) and in SPF mice at their arrival (0.1–1.8%) (Figure 4F). Age and initial microbiota depletion using laxatives or antibiotics had no impact on the rise in *Blautia* 3 weeks after mice arrival. However, GF recipients were significantly less affected by this *Blautia* overabundance as this genus represented only 0.28 and 3.48% of the microbiota 3 and 9 weeks after colonization.

### Germ-Free Period Has Long Lasting Effect on the Gut Physiology

**FIGURE 5:**
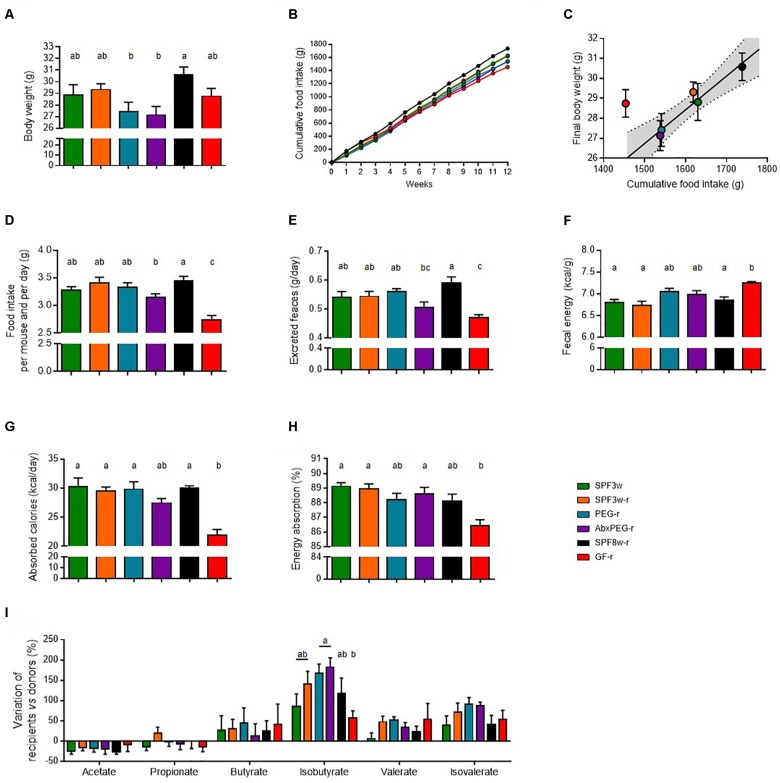
Recipient age, microbiological status, and pre-treatment before microbiota transfer and influence on digestive physiology. **(A)** Body weight at the end of the experiment. **(B)** Cumulative food intake. **(C)** Food intake 10–12 weeks after fecal microbiota transfer. **(D)** Feces output per day and per mouse. **(E)** Fecal energy, expressed as calories per gram of dry feces. **(F)** Energy absorption, expressed as calories per day and per mouse. **(G)** Energy absorption capacity, expressed as the percentage of total energy ingested. **(H)** Cecum weight. **(I)** Variation of the concentration short-chain fatty acids in the cecal content of recipient mice in comparison with donor mice. Mice groups with different letters are significantly different (adjusted *p* < 0.05).

Given the importance of the intestinal microbiota on energy extraction and absorption, we measured body weight and food intake during the whole experiment as well as energy absorption during 3 weeks, starting 9 weeks after *Lep^ob^* microbiota inoculation (Figure 1A and Figure 5).

Both SPF mice that arrived when weaning and GF mice had similar final body weight whereas SPF8w-r tended to weigh slightly more than both groups at the end of the experiment (Figure 5A). In SPF mice, linear regression with a 95% confidence interval indicated that the final body weight was associated with the cumulative food intake at the end of the experiment (Figures 5B,C), but this was not the case for GF-r mice. GF-r recipients had the lowest cumulative food intake but displayed a similar weight as SPF mice. The GF-r group consumed the same amount of food as their SPF8w-r age-matched counterparts during the first two weeks following colonization but then started to eat significantly less than the other groups (Figure 5B). This resulted in a lower cumulative food intake in GF-r mice. In comparison to weight-matched mice, GF-r excreted a smaller amount of feces but these feces contained significantly more kilocalories per gram (Figures 5E,F). Importantly, this resulted in less absorbed energy per day and a significantly lower net intestinal absorption capacity (Figures 5G,H). Likewise, AbxPEG-r mice tended to have a lower food intake and absorb less energy than SPF3w-r and PEG-r groups, although these changes were much more moderate than in GF-r and did not reach statistical significance.

Production of short chain fatty acids (SCFA) from non-digestible compounds is one of the mechanisms by which the gut microbiota is able to modulate the amount of energy absorbed from the food (Bergman, 1990). We measured the concentration of SCFA in the cecal content of donor and recipient mice. The concentration of the SCFAs in each recipient group was expressed in proportion (%) to the mean concentration in the cecal content of donors (Figure 5I). The concentration of acetate, propionate, butyrate, valerate and isovalerate was similar in all recipient groups while the concentration of isobutyrate was higher in the cecal content of PEG-r and AbxPEG-r groups compared to GF-r mice. This SCFA accounted for less than 0.6% of the total SCFA amount and did not cause differences in the total amount of SCFAs. The lower capacity of energy absorption of GF-r mice is likely not the consequence of a less efficient energy extraction from the food through fermentation by the gut microbiota.

## Discussion

Transmission of donors phenotypes in recipient hosts by intestinal microbiota transfer is critical to demonstrate the causal role of a given intestinal microbiota in physiological and pathological processes (Fritz et al., 2013). GF mice, SPF mice with or without prior intestinal microbiota depletion are frequently used as recipient models; however, the specificities of each model are still poorly described. In particular, precisely assessing the engraftment dynamics of the donor microbiota is necessary. We therefore described the impact of murine recipients’ microbiological status, age, and prior endogenous microbiota depletion on fecal microbiota transfer efficacy. We tested the effect of recipient model choice in the specific context of *Lep^ob^* to wild-type mice gut microbiota transfer, nevertheless our results suggest that simple gavage administration of mouse donor microbiota effectively reshapes intestinal microbiota in SPF recipients. In agreement with previous studies (Schloss et al., 2012; Zhang et al., 2014; Rausch et al., 2016), aging and husbandry conditions have a significant impact on engraftment dynamics and microbiota composition.

Some microbial compositional features in recipient mice did not depend on the model. For example, lactobacilli were less abundant in all models of recipient mice than in the donor inoculum. It can be hypothesized that these microbial features are due to host genotype, i.e., leptin deficiency of *Lep^ob^* mice in our experiments, and thus not transferable in wild type recipients.

Importantly, we observed that the presence of endogenous microbiota had little barrier effect against exogenous taxa gut colonization. Nevertheless, the removal of endogenous taxa, so that the recipient’s microbiota more closely resembles donors’ microbiota, was more effective in weaning mice than in adults. The gut microbiota has been reported to be less diverse at weaning (Zhang et al., 2014), and intestinal microbiota transfer has been shown to be more efficient if the recipient’s microbiota exhibits lower richness (Ericsson et al., 2017). Likewise, in human fecal microbiota transfer (Kootte et al., 2017), a beneficial phenotype was transferred solely in receivers with low richness, yet the authors did not evaluate the similarity between the donor inoculum and microbiota in recipients. Our work shows that microbiota engraftment performed better in younger mice, although we did not observe any differences in microbiota richness in adult and 3 week old C57BL/6J mice. Thus, low microbiota richness was not a determinant to explain the better microbiota engraftment in juvenile mice in the present study.

As previously shown in adult mice, we hypothesized that endogenous microbiota depletion using laxatives in juvenile mice would allow the persistent removal of a greater number of endogenous taxa. Bowel cleansing with PEG proved efficient as more endogenous taxa were eliminated, but this treatment had little impact on bacterial taxa proportions. The intestinal microbiota is resilient and recovers from bowel cleansing within 2–4 weeks in humans (Jalanka et al., 2015), an observation that can likely be extended to rodents. Bacteria adhering to the mucus and not flushed out by the laxatives, which might be sufficient to rescue the initial intestinal microbiota.

Whereas high doses of broad spectrum antibiotics deplete intestinal microbiota (Reikvam et al., 2011), residual antibiotics might persist in the mice and have deleterious consequences on the implantation of inoculum’s bacteria. We thus subjected antibiotic-treated mice to bowel cleansing with PEG before *Lep^ob^* microbiota inoculation. Quite surprisingly, not only did this approach have very little impact on the number of successfully removed endogenous phylotypes, but it altered the colonization dynamics of the inoculum. We observed a transient overproliferation of *Parasutterella*, *Coriobacteriaceae*, bifidobacteria and unknown *Gastranaerophilales* three weeks after the colonization and these changes disappeared 6 weeks later. Moreover, the implantation of *Lachnospiraceae*, *Oscillibacter* and unclassified bacteria, among others, was delayed. Our observations did not support the hypothesis that the partial or complete depletion of initial gut bacteria communities with antibiotics would significantly enhance the fecal microbiota transfer efficacy in mice when administrated in young animals.

The optimal SPF recipient model from these results appears to be weaning mice subjected to bowel cleansing with PEG prior to microbiota transfer. This finding is not in line with previous reports stating that prior antibiotic treatment allows a better engraftment than bowel cleansing alone (Ji et al., 2017). However, in their study, Ji et al. (2017) used 50 mg of PEG to clean the bowel of adult mice, which very likely weighed at least 22 g, while we used 93 mg of PEG to clean the bowel of young mice, weighing between 8 and 11 g. The higher dose of PEG used here probably allowed a more efficient bowel cleanse, as it is supported by recent findings reporting that at least 170 mg of PEG are necessary to obtain significant bacterial load reduction in adult mice (Wrzosek et al., 2018).

Here, SPF mice colonized at weaning performed as well as GF mice, which are currently the gold standard model proposed in fecal microbiota transfer studies. Nine weeks after the microbiota transfer, the number of phylotypes detected in recipients but not in the inoculum was 73 in PEG-r mice and 80 in GF-r mice. These unexpected phylotypes constituted a moderately lower proportion in PEG-r microbiota than in GF-r microbiota, both short- and long-term after microbiota transfer. More importantly, the ecosystem structure in GF recipients 21 days after colonization was altered and did not resemble donor inoculum, non-inoculated SPF microbiota, or SPF recipients. At the phylum level, Bacteroidetes, Actinobacteria and Verrucomicrobia phyla were significantly enriched while Firmicutes were depleted. Additionally, GF recipient mice displayed half the bacterial richness compared to all other recipient groups. Six weeks later, at the 9 weeks post-inoculation time-point, the abundance of Bacteroidetes, Actinobacteria and Verrucomicrobia phyla decreased and was similar in GF-r and in the inoculum. This was associated to a significant increase of Firmicutes abundance. Additionaly, bacterial richness increased and PEG-r and GF-r microbiota displayed the same degree of similarity with the inoculum 9 weeks after microbiota transfer. Peculiar features associated with SPF mice treated with antibiotics were observed at least to the same extent in GF recipients. Thus, delayed establishment of unclassified bacteria and *Oscillibacter* genus together with a transient overproliferation of *Parasutterella* genus and Actinobacteria phylum result likely from a common characteristic of both models: the establishment of bacterial communities in an initially GF or nearly GF gut, rather than from components specific of each model, like the presence of residual antibiotics or an immature mucosal immune system.

The different colonization dynamics of the GF gut has already been observed. This finding suggests that colonization is characterized by transitory stages with bacterial communities adapting to the host physiological changes, which precedes the stabilization and convergence toward the inoculum community (Gillilland et al., 2012; Tomas et al., 2013). Here, our results suggest that this stabilization is reached between 3 and 9 weeks after microbiota transfer in GF mice, which should be taken into consideration for planning experimental designs and interpreting results related to phenotype transmission. Indeed, numerous studies using GF mice last less than a few weeks, at a time when the intestinal ecosystem is still evolving and distant from the inoculum, which could lead to improper conclusions. Our current results favor to verify whether the transmitted phenotype in these short-term studies is still present at a later time point, when the composition of the recipient microbiota has stabilized.

In addition, we found that unclassified bacteria are depleted during the first few weeks of the ecosystem establishment. The fact that these bacteria are unknown suggests that they are resistant to current cultivation techniques, which indirectly indicates that they require specific survival and growth conditions. The physicochemical conditions in the GF gut are different from the conventionalized gut. Specifically, increased pH, increased concentration of urea and oxygen, and absence of SCFAs (Al-Asmakh and Zadjali, 2015) are among the numerous characteristic differences that can have a significant impact on microbiota engraftment. The bacteria that can tolerate these conditions are thus favored during the early stages of the colonization to the detriment of more sensitive taxa, such as unclassified bacteria.

Other transient microbiota compositional features were specific to GF recipients, indicating that these features were the consequence of GF status during host development rather than short-term microbiota depletion pre-inoculation. Three weeks after the inoculation, GF recipients were mainly characterized by a tremendous enrichment in *Ruminococcus gauvreaui*, *Akkermansia* and *Bacteroides* genera. GF development also leads to numerous functional, metabolic, and immunological alterations (Desbonnet et al., 2013; Smith et al., 2017), most of which are reversible by conventionalization. However, this “normalization” of the GF physiology does not occur instantaneously, which may differentially shape bacterial communities in former GF mice relative to SPF mice. In particular, the immune system of GF mice is immature and the induction of innate and adaptative immune functions in the intestine are rapidly initiated but requires 30 days to be completely functional (El Aidy et al., 2012, 2013). The transient lack of pressure exerted by the immune system on the intestinal microbiota, by the way of secreted IgA and antimicrobial peptides in particular, could explain the overproliferation of certain bacterial taxa at the 21 days post-inoculation time point. This is likely the case for the *Akkermansia* genus, a taxa which is significantly increased in immunodeficient *Rag1^-/-^* mice (Zhang et al., 2014). Overall, experimental designs combining GF recipients together with microbiota composition analysis and phenotype characterization less than 3–4 weeks after inoculation potentially prevent unexpected findings, such as the impact of unclassified bacteria on host health.

Similar to the immune system, functional maturation of the gut epithelium following colonization is not instantaneous. For example, functional maturation of the colonic epithelium takes at least 2 weeks and the formation of a proper mucus layer requires 5–6 weeks (Tomas et al., 2013; Johansson et al., 2015). Here we report that basic gut functions such as energy absorption are still diminished 9–12 weeks after conventionalization of 8 week-old GF mice in comparison with SPF mice. Despite eating less food and having a lower energy absorption, conventionalized GF mice still had a similar weight compared to SPF counterparts, suggesting that not only digestive system function, but also overall energy metabolism is impaired in the longer term.

## Conclusion

We report that SPF mice colonized with *Lep^ob^* mice microbiota at weaning after initial microbiota depletion with laxatives or antibiotics perform as well as GF mice recipients in experiments lasting at least 9 weeks. Our results also demonstrate that bowel cleansing with PEG allows proper endogenous microbiota removal and inoculum microbiota implantation and that antibiotics do not provide any further improvement. Whereas none of the tested models allow perfect replication of the inoculum microbiota composition, GF mice might be not the optimal model for short-term experiments, at least not until a homeostatic state is achieved. Additional experiments with microbiota donors other than genetically obese mice are needed to evaluate the versatility of juvenile SPF mice treated with high-doses of laxatives as recipient model. Nevertheless, the present study suggest that it constitutes a valid alternative model to GF mice when GF mice of a specific strain or GF facilities are not available. In order to establish the translational value of this model, further studies are needed to determine whether weaning SPF mice combined with microbiota depletion are as effective in engrafting human microbiota communities as they seem to be for murine microbiota.

## Ethics Statement

This study was carried out in accordance with the recommendations of the Directive 2010/63/EU of the European Parliament and of the Council of 22 September 2010 on the protection of animals used for scientific purposes. The protocol was approved by the “Comité d’éthique en expérimentation animale Charles Darwin n°5” and received permission number 01746.01 from the “Ministère de la Recherche”.

## Author Contributions

KC is the coordinator of Metacardis consortium. TLR and KC designed the study. TLR, JD, CD-C, FI, and FM carried out the experiments. TLR and JD analyzed the data and performed the statistical analysis. TLR, JD, MG-M, NK, JA-W, and KC contributed to data interpretation. TLR, KC, and JD wrote the first draft of the manuscript. All authors contributed to the writing and approved the final version of the manuscript.

## Conflict of Interest Statement

The authors declare that the research was conducted in the absence of any commercial or financial relationships that could be construed as a potential conflict of interest.
