# Supplementary figures and images for: Comparative Evaluation of Microbiota Engraftment Following Fecal Microbiota Transfer in Mice Models: Age, Kinetic and Microbial Status Matter
Source: Front Microbiol. 2019 Jan 14;9:3289. doi: 10.3389/fmicb.2018.03289 (PMC6339881; doi:10.3389/fmicb.2018.03289)

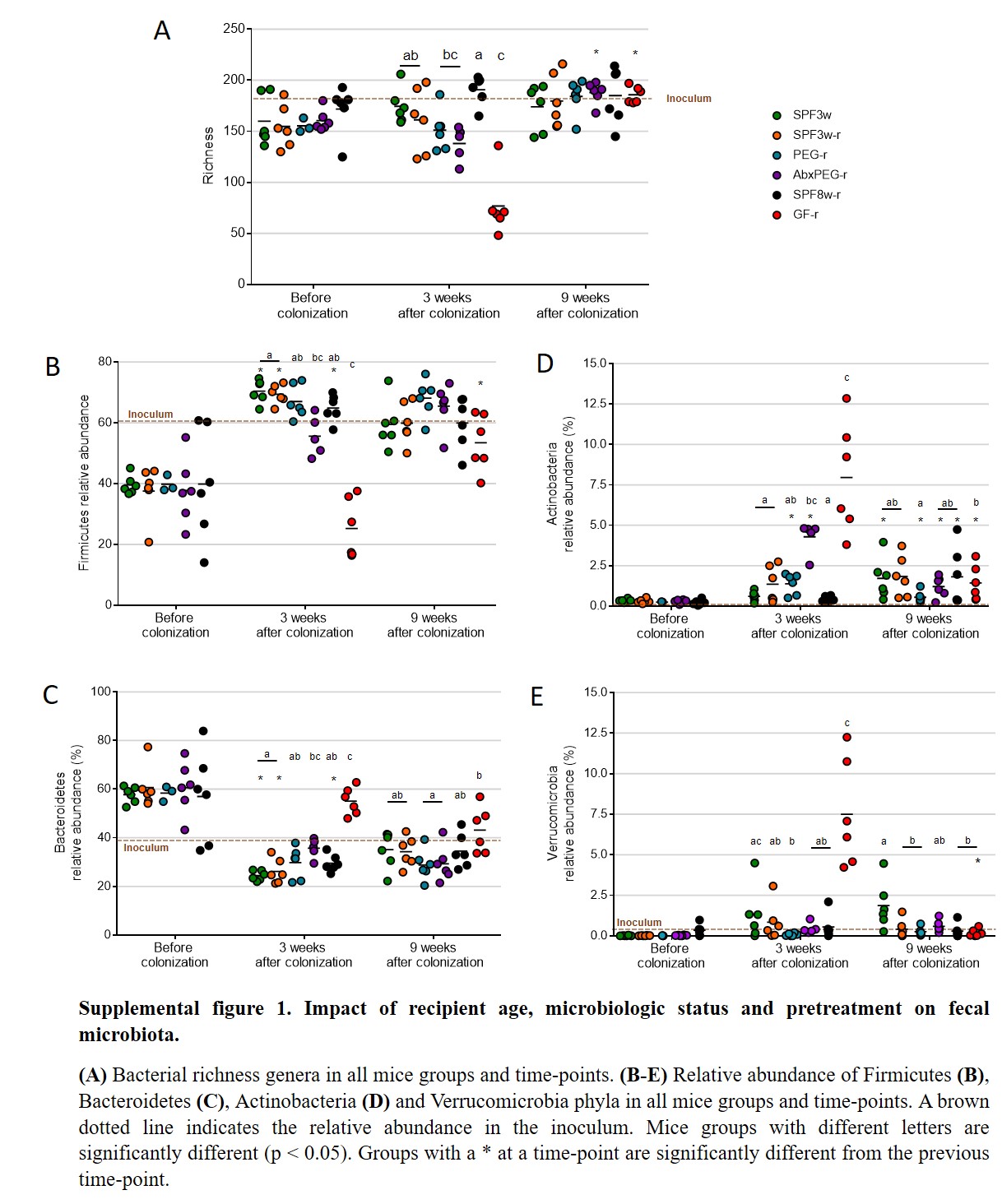

Supplement: Supplementary file 1 [file Image_1.jpg]

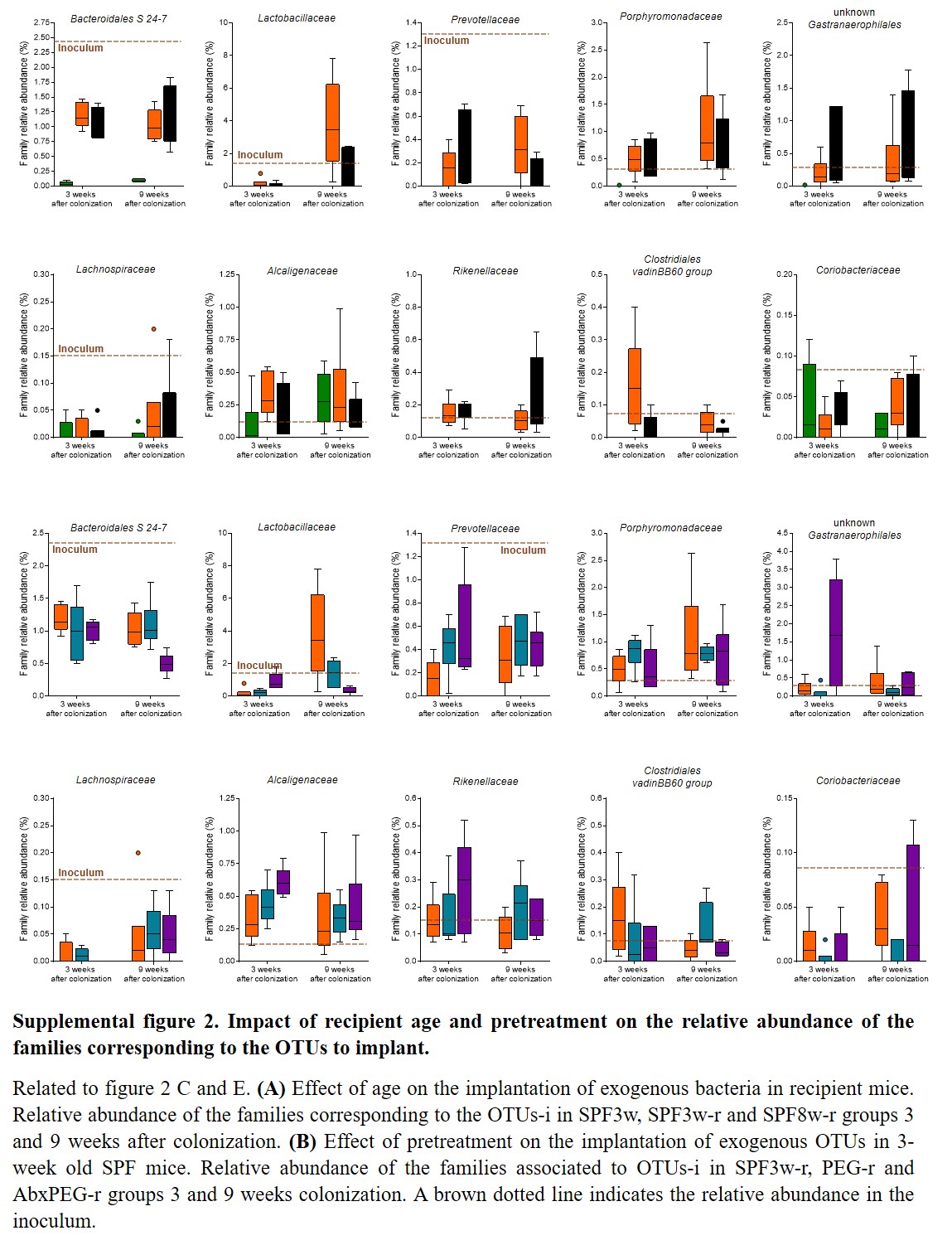

Supplement: Supplementary file 2 [file Image_2.jpg]

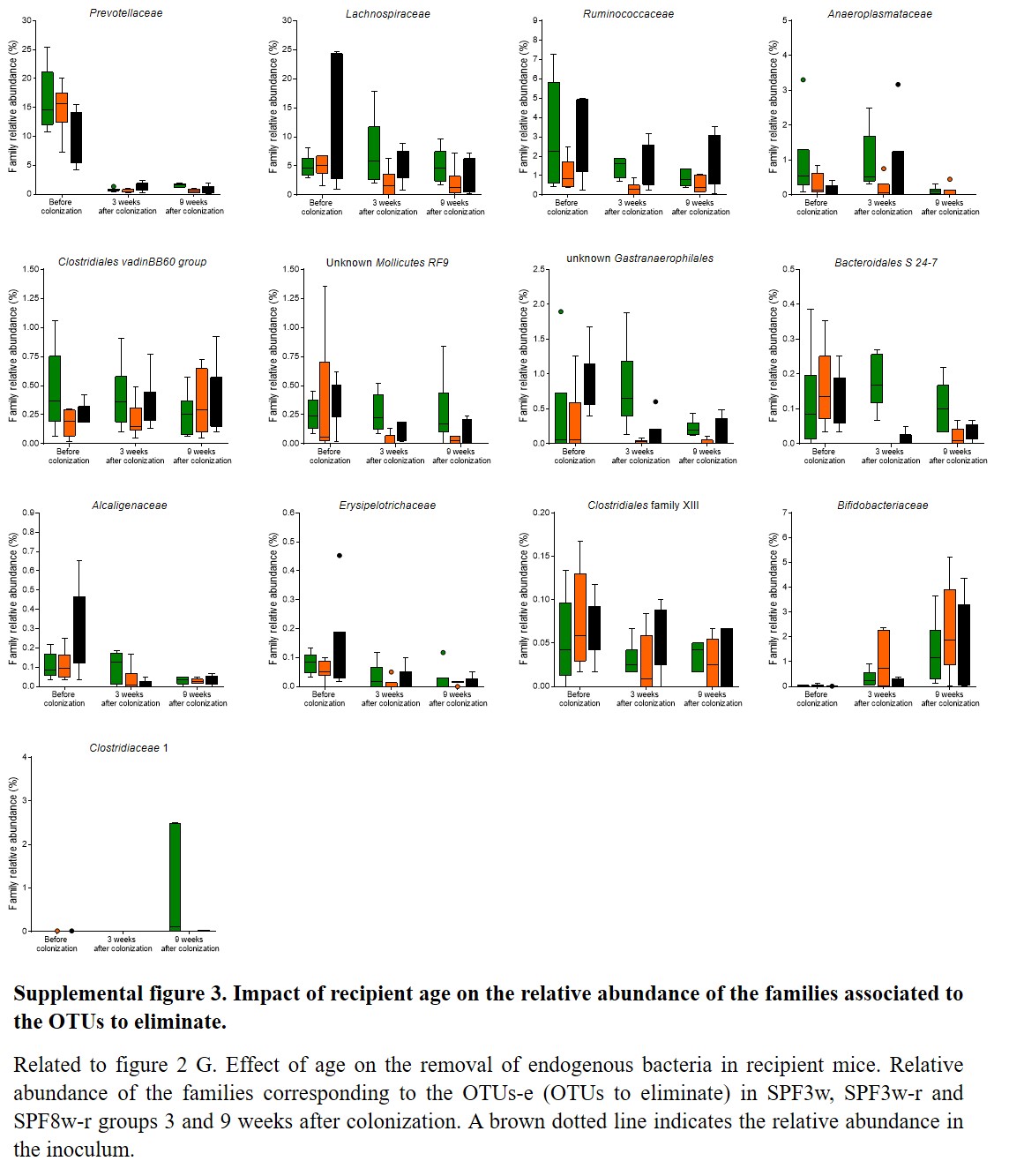

Supplement: Supplementary file 3 [file Image_3.jpg]

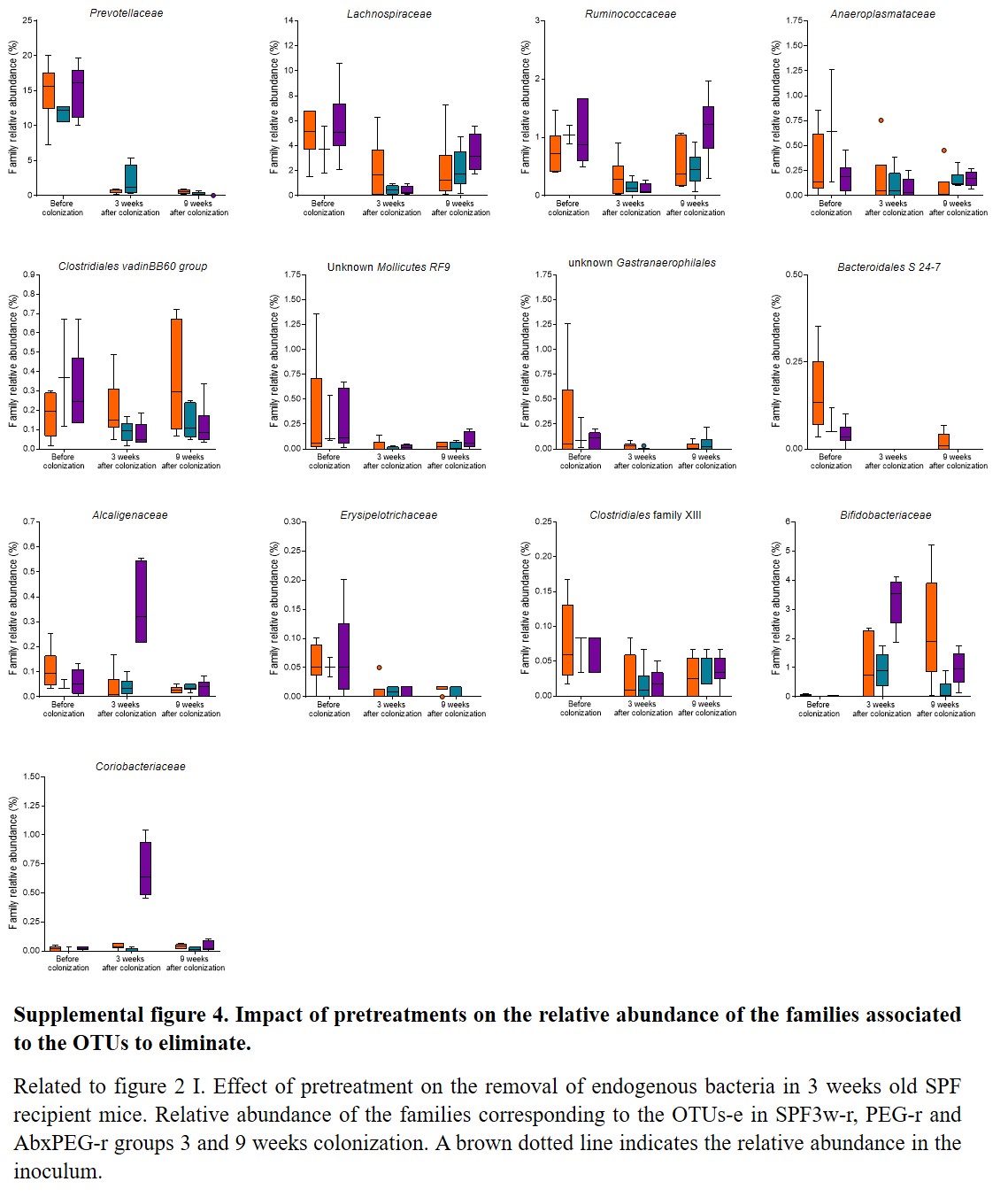

Supplement: Supplementary file 4 [file Image_4.jpg]

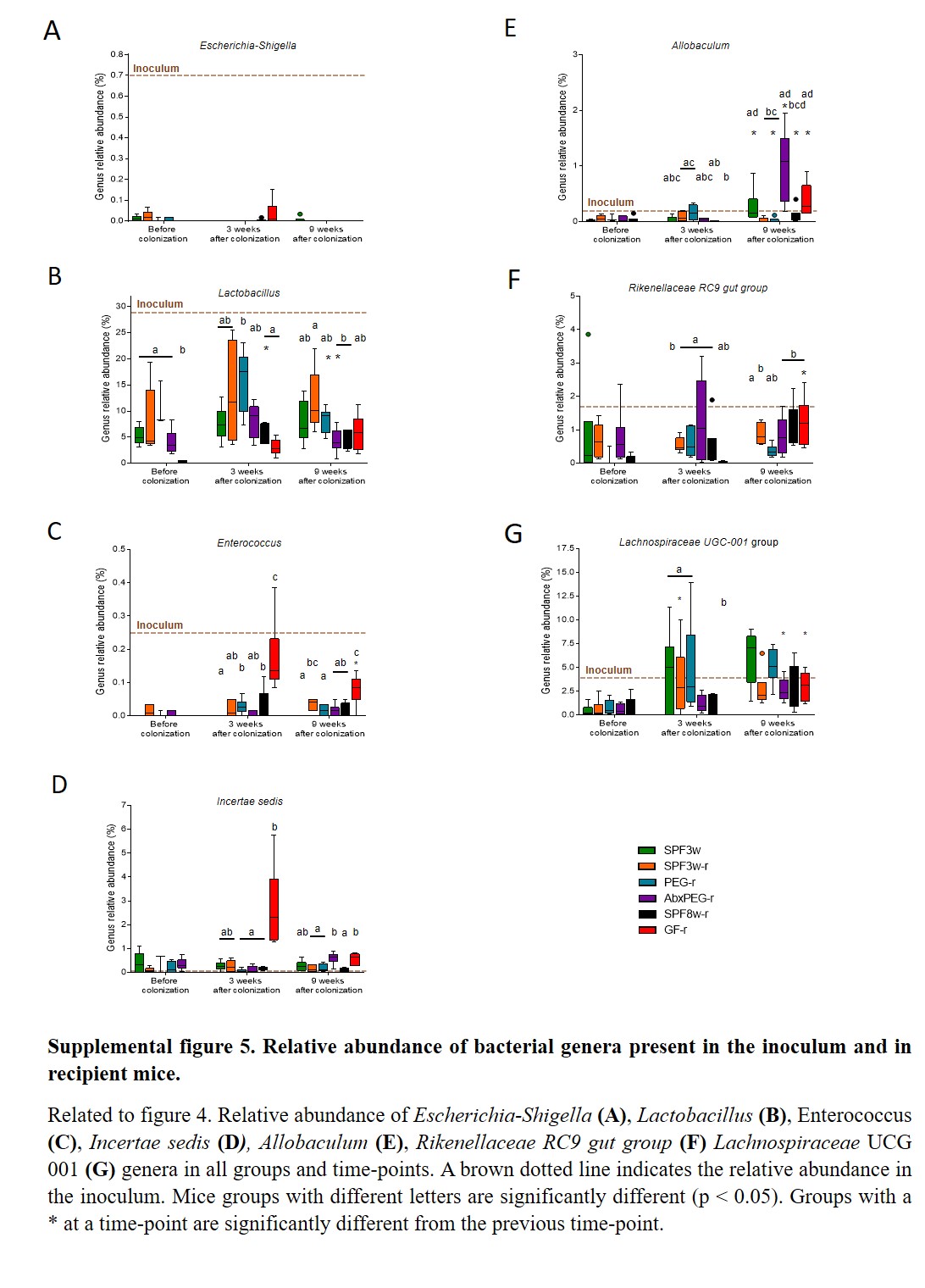

Supplement: Supplementary file 5 [file Image_5.jpg]
